# Monitoring temporal changes in large urban street trees using remote sensing and deep learning

**DOI:** 10.1371/journal.pone.0326562

**Published:** 2025-06-26

**Authors:** Luisa Velasquez-Camacho, Natalie van Doorn, Haiganoush Preisler, Maddi Etxegarai, Oriol Alas, Jose M. Gonzalez Castro, Sergio de-Miguel

**Affiliations:** 1 Department of Plant Sciences, University of California Davis, Davis, California, United States of America; 2 Eurecat, Centre Tecnològic de Catalunya, Unit of Applied Artificial Intelligence, Barcelona, Spain; 3 USDA Forest Service, Pacific Southwest Research Station, Albany, California, United States of America; 4 Department of Agricultural and Forest Sciences and Engineering, University of Lleida, Lleida, Spain; 5 Forest Science and Technology Centre of Catalonia (CTFC), Solsona, Spain; Universidad Tecnica Federico Santa Maria, CHILE

## Abstract

In the rapidly changing dynamics of urbanization, urban forests offer numerous benefits to city dwellers. However, the information available on these resources is often outdated or non-existent, leading in part to inequitable access to these benefits for the population. Access to equitable and just green spaces is a challenge for local governments, allowing the city’s inhabitants to enjoy a healthy environment. In this context, remote sensing serves as a powerful data source that enables the monitoring of the evolution and dynamics of cities over time, as well as changes in urban forests. For this study, our focus is on large trees, defined as those with a canopy diameter exceeding seven meters. These trees play a vital role in offering ecosystem services that improve the environment, biodiversity, and mental health of the inhabitants of cities. Using deep learning algorithms, we identify the large urban street trees in National Agriculture Imagery Program (NAIP) images and analyze the changes in large street trees over an 18-year period (2005–2022) in six counties of the San Francisco Bay Area. We successfully tracked changes in the presence of large trees in the public right-of-way at the census tract, city, and county levels. We tracked changes in large tree availability at the neighborhood, city, and county levels, revealing socio-demographic disparities. Our analysis found that census tracts with higher household incomes, a greater proportion of individuals who self-identify as white, and more families were positively associated with an increase in large tree canopy. This assessment provides insight into the varying levels of access to ecosystem services offered by large trees across urban environments.

## Introduction

Urban forests have been extensively researched and found to provide numerous benefits to cities, significantly improving the quality of life of their inhabitants. In this regard, urban trees play a crucial role in guaranteeing equitable access to ecosystem services (ES). Street trees are especially important in cities and towns with high building density where the yard space is limited, as they are often the only opportunity for planting and greening. Additionally, they are often the only green space residents can enjoy [1,2]. Due to their multiple ecosystem functions, these trees are an essential part of the urban forest [3]. Their high visibility and aesthetic value make them frequent targets of planting and management programs by municipal arborists [4]. They are also a valuable asset in urban design, effectively addressing a range of climate-related issues [5]. The natural ability of local street trees to provide ecosystem services, such as cooling microhabitats and providing shade, has been highlighted in studies by Anderson et al. [6] and Salmond et al. [5].

Moreover, studies have shown a direct connection between the presence of urban street trees and socioeconomic factors, such as housing prices [7,8] and neighborhood categorization. Neighborhoods characterized by lower and insufficient biodiversity in their street trees typically correspond to D-graded (based on the classification of HOLC (The Home Owners’ Loan Corporation) grade assignment [9] or segregated neighborhoods [10] in the United States. It is well documented that neighborhoods with higher incomes are at lower risk of death from air quality-related causes [11] where good access to green spaces is common [8,12]. Another finding is that neighborhoods with higher tree density experience lower crime rates [13–16]. According to these studies, green spaces can serve as a deterrent to crime and enhance urban areas’ safety and security [17]. High-quality landscaping around a home can deter criminal activity by giving visual signals to potential criminals or intruders that residents take good care of their territory [18]. However, it is essential to acknowledge that there may be disparities in access to these benefits [10].

This growing awareness has produced discussions around the equitable distribution of trees, forests, and green spaces in urban environments, with the 3-30-300 rule gaining importance in 2022 [19,20]. This rule determined that each household should have access to a minimum of 3 large trees visible from their home windows, that every neighborhood should maintain a canopy cover of at least 30%, and that green spaces should be located within 300 meters of every house [19]. The 3-30-300 rule underscores the importance of ensuring access to urban green spaces for all communities, regardless of socioeconomic status [12,21].

In this perspective, the role of large and mature street trees becomes pivotal in achieving green justice (GJ). Large trees have the potential to offer a greater range of ecosystem services compared to smaller and recently planted trees [22]. These benefits have been also well-documented, particularly in their high capacity for carbon sequestration [23,24], provision of shade [25], maintenance of ecosystem resilience [26,27], and the provision of habitat for a diverse number of species, including birds, invertebrates, and other plants like bromeliads [27–30]. For this study, we define large street trees as those with a canopy section exceeding 64 m^2^ or 7 meters in any cardinal direction. Even with their socio-ecological significance, the management of large urban street trees often has complex challenges in urban planning and design [1,31]. These trees can lead to conflicts that may result in their removal, often being replaced with smaller and younger trees [31–34], or they could be completely eliminated to satisfy the growing infrastructure demands of urbanizing cities. Our focus was specifically on large street trees, as they fall under the jurisdiction of local governments, and their changes can serve as indicators of urban dynamics and management policies [23,24,35,36]. Regulations regarding street tree removal and planting in the San Francisco Bay Area are governed primarily at the city level, with local ordinances specifying the permit process, conditions for removal, and requirements for tree replacement.

Addressing issues of green space access and the advantages offered by urban forests, and especially by large urban trees, has become increasingly crucial, yet the analysis of spatial and temporal patterns of individual trees over time remains sparse. By 2020, only 13% of cities in California (CA) had a forest inventory [37,38], and urban forest inventories are even more rare in other parts of the world. Furthermore, when such inventories do exist, they often represent a single data point, making it challenging to track changes over time. In general, the scarcity of data makes it difficult to discern disparities in green space access and understand the distribution and impact of urban trees in urban areas worldwide [39].

Nevertheless, increased computational power and the availability of remote sensing data have played a crucial role in enhancing the identification of objects such as street trees [40,41]. Specifically, advancements in remote sensing data allow for deeper analysis, facilitating multitemporal assessments to track changes over time. Within this framework, artificial intelligence algorithms have introduced innovative methods for more efficient extraction of data from remote sensing images [42,43]. This capability enables their application and scalability to extensive datasets, making them suitable for deployment across expansive geographical areas [44]. This represents an effective solution for governmental entities with limited budgets seeking to generate data for urban trees.

In this study, we employed a deep learning (DL) model to map and track changes in large street trees using the San Francisco Bay Area (referred to hereafter as the Bay Area) as the study area. Using National Agriculture Imagery Program (NAIP) orthophotography spanning seven years between 2005 and 2022, we mapped these trees and conducted a detailed analysis of their dynamics. Our investigation focused on various spatial scales, based on census tract identification (IDs) including blocks, places, and county levels. In this study, we frame urban street trees as socio-ecological infrastructure whose distribution can reflect patterns of environmental justice (EJ). We interpret variables such as large street tree density per capita, canopy cover, household income, and racial composition as environmental justice indicators that allow us to assess the unequal distribution of green infrastructure and its potential implications for historically disadvantaged communities. We addressed the following research questions (RQs):

- How do the spatial distribution and temporal changes of large street trees vary across census tracts, neighborhoods, and counties in the San Francisco Bay Area from 2005 to 2022?
- What is the relationship between changes in large street tree cover and socio-demographic variables?
- To what extent do disparities in large street tree canopy correlate with indicators of EJ, and how do these patterns change over time?

## Materials and methods

### Study area

**Fig 1:**
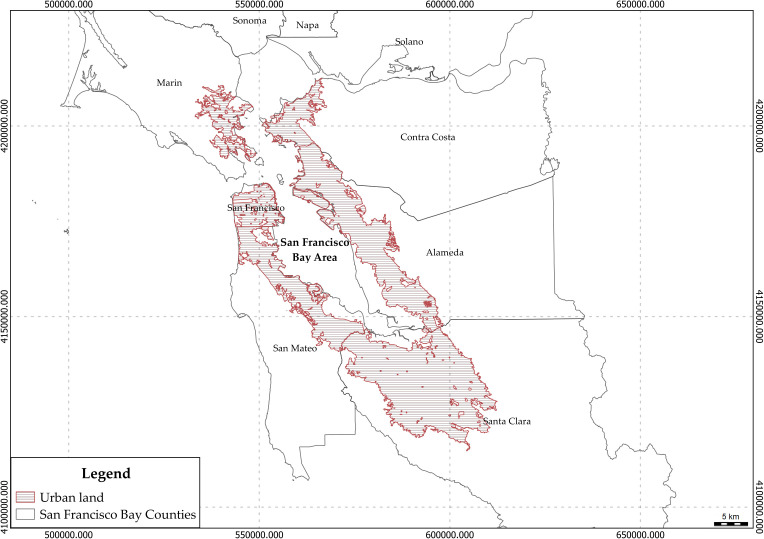
Study area. Urban lands in the San Francisco Bay are represented by the red polygon.

The study area encompasses the urban land in the San Francisco Bay Area in northern California, USA (Fig 1). The San Francisco Bay Area has an area of approximately 18,000 km^2^, of which only approximately 3,000 km^2^ are considered urban areas [45]. This area encompassing counties such as Alameda, Contra Costa, Marin, Napa, San Francisco, San Mateo, Santa Clara, Solano, and Sonoma, is notably recognized as the fourth most densely built urban area within the United States [46–48]. With a population exceeding seven million residents and an urban population density of 3,000 individuals per square meter [49], this region has been undergoing a rapid process of urbanization and gentrification. Among the counties within the Bay Area, Santa Clara County stands as the largest, encompassing the city of San José, with a population of approximately 1.9 million. Following closely, Alameda County (with its city of Oakland) has a population of around 1.6 million, and Contra Costa County has approximately 1.1 million residents. The remaining counties have populations ranging from 800,000–400,000 individuals [50]. In 2007, it was reported that this region hosted a considerable number of urban trees, reaching a total of approximately 41 million [51].

San Francisco Bay area has a coastal Mediterranean climate marked by a dry summer and fall season followed by a wet winter. However, diverse geographic features, ranging from mountain ranges to inland valleys and smaller bays, form several distinct climatic zones [52]. This ecological richness is sustained by almost 500 continuous large, protected areas [53]. Additionally, the Bay hosts 90 percent of California’s remaining wetlands.

#### Socioeconomic description.

The San Francisco Bay Area is characterized by significant socioeconomic diversity and disparity. An influx of high-wage tech employees, a booming tourism sector, and limited housing availability have driven rapid urbanization and gentrification in the Bay Area [54,55]. This has led to a considerable increase in housing costs, contributing to displacement and socioeconomic stratification within the region. Local households spend on average 48% of their income on housing, compared to the national average of 29%. Only 12% of residents can afford a median-priced home, and a quarter of Bay Area renters are considered severely housing-burdened, spending more than 50% of their income on housing costs [56].

Wealthier communities, such as Palo Alto and Menlo Park, boast some of the highest median household incomes in the country, while lower-income areas like Oakland and Richmond struggle with higher unemployment rates and reduced access to quality housing and public services [57,58].

Gentrification in the Bay Area is closely associated with displacement, particularly impacting low-income and minority communities. According to the Urban Displacement Project [59], less than 10% of all tracts in the Bay Area are classified as undergoing early or advanced gentrification. However, around 30% of all tracts are at risk of becoming exclusive or are already stable/advanced (the neighborhood has been high-income or mixed high-income since 2000 and show patterns of exclusion [59]) exclusive, posing accessibility challenges for low-income households.

This socioeconomic change has significant implications for environmental and greening justice. Wealthier neighborhoods often benefit from better-maintained green spaces and higher tree canopy coverage [6], which contribute to improved air quality, lower urban heat island effects, and enhanced mental well-being. In contrast, lower-income areas often lack sufficient green spaces and urban tree coverage [60]. This disparity makes worse environmental inequities, as these communities are more exposed to environmental problems such as air pollution and extreme heat, while also being less resilient to the impacts of climate change.

West Oakland exemplifies these challenges [61]. Historically it is one of the poorest neighborhoods in the Bay Area. Also, it has the highest concentration of air pollutants in the Bay Area and has experienced significant displacement of African American residents since 1990 [62,63]. The environmental impact of gentrification, such as expulsion, displacement, and community fragmentation are disproportionately borne by low-income, minority-dominated areas like West Oakland [64].

These patterns are also evident in the distribution of urban forest cover in San Francisco. For instance, South of Market and Downtown/Civic Center, areas with high poverty rates and significant racial minority populations [65], have the lowest large street tree density. In contrast, the Seacliff neighborhood, one of San Francisco’s wealthiest districts, has the highest density of trees outside of parks [66].

To study the patterns and distribution of large street trees as an indicator of greening justice, we selected six counties: Alameda, Contra Costa, Marin, San Francisco, San Mateo, and Santa Clara. These counties collectively form a contiguous urban area and encompass 58 cities and 1104 Neighborhood Tabulation Areas (NTAs) within their urban zones. NTAs, recognized as medium-sized statistical geographic units, serve as the framework for reporting data from the Decennial Census and the American Community Survey (ACS), providing valuable insights into the demographic and socioeconomic characteristics of these areas.

### Data

#### Aerial images.

In this study, the NAIP was utilized, which provides digital orthoimages consisting of four bands (Red, Green, Blue, and near-infrared) available only in the United States [67]. These orthoimages are captured specifically during the growing season, offering valuable data for vegetation and land cover analysis [68,69]. The day of the year (DOY) was determined through metadata consultation. In most years, the images were captured during different DOY corresponding to different seasons. One image per county was obtained from the official USDA data Geohub (https://nrcs.app.box.com/v/naip), starting from the year 2005–2022. A total of 42 images were downloaded and processed (Table 1).

**Table 1:** Spatial and temporal resolution of NAIP images consulted in the study area. DOY: Day of Year.

Year	Resolution (m)	DOY (Ordinal Day)
2005	1	163-229
2009	1	159-187
2012	1	141
2016	0.6	150-177
2018	0.6	204-218
2020	0.6	145
2022	0.6	139

To overcome compatibility issues related to the proprietary format of NAIP images (MrSID), a series of preprocessing steps were implemented. These steps involved converting the images to the.tiff format, followed by cropping to focus specifically on the urban areas of interest. Additionally, compression techniques were applied to optimize the file sizes while preserving crucial information.

#### Socioeconomic data.

We used data from the Urban Displacement Project collected by the University of California Berkeley and the University of Toronto [59]. This project aims to map patterns of socioeconomic change at the tract level, with a particular focus on variables related to processes of gentrification, displacement, and exclusion in cities worldwide. The database comprises approximately 305 variables, containing historical information from 1990 to 2018 in the entire San Francisco Bay Area. We focused on 17 specific variables from this database (Table 2) that allowed us to find the relationship between population dynamics, economic development, and changes in the presence of large trees over time as an indicator of greening justice.

**Table 2:** Explanatory variables analyzed to model the effect on the rate of change on large tree presence and large trees per capita and canopy cover in large trees between 2005 and 2022. The socioeconomic variables were acquired in the Urban Displacement Project (Chapple et al., 2021).

Variable	Unit
Canopy Area of Large Trees	m^2^
Cover Area of Large Trees	%
Large Tree Density	Number
Trees In 2005	Number
Cover Area of Large Tree Canopy	km^2^
Image Spatial Resolution	Pixel size
DOY	Ordinal day (Table 1)
Total Population	Number
Tree Per Capita	Rate
Population Density	Number
Proportion of White Residents	Number
House Income	USD
Family Density	Number
Dense tracts	Binary
Advance Exclusive	Binary
Stable Advanced Exclusive Typology	Binary
Advance Gentrification	Binary
At Risk of Being Exclusive	Binary
Become Exclusive	Binary
Stable Middle/Moderate Income	Binary
At Risk of Gentrification	Binary
Early Ongoing Gentrification	Binary
Ongoing Displacement	Binary
Advance Exclusive	Binary

### Methods

In this study, we utilized a retrained Convolutional neural network (CNN) called You Only Look Once V5 (YOLO) model trained by [43] for the detection of urban tree canopies in high-resolution aerial and satellite RGB images. YOLO is a state-of-the-art object detection method that identifies and locates objects in images in a single step, significantly reducing analysis time (Ponnusamy et al., 2020). The model was retrained using 100 high-resolution NAIP images from several cities in California, Utah, Kentucky, and Louisiana, USA. This retraining was necessary because the original model had been trained on higher-quality images with a resolution of 0.15 to 0.60 meters. The training process involved 50 epochs, allowing the model to learn and accurately identify large trees in these images. To evaluate accuracy, precision, and recall, we used the values from the confusion matrix. This matrix consists of True Positives (TP), True Negatives (TN), False Positives (FP), and False Negatives (FN). TP denotes large trees accurately identified by the model. TN signifies non-tree objects, such as background elements, correctly identified. FN represents large trees present in the ground truth but missed by the model. Lastly, FP accounts for objects wrongly classified as trees by the model, when they are not [70].

(a) Accuracy = (TP + TN)/ (TP + TN + FP + FN)
(b) Precision = (TP)/ (TP + FP)
(c) Recall = (TP)/ (TP + FN)

We adjusted the image size according to the recommendations of Velasquez-Camacho et al. (2023) to improve the detection capacity of their model. Specifically, we chose a window size of 250 pixels by 250 pixels for images with a spatial resolution of 1 meter and 350 pixels by 350 pixels for images with a spatial resolution of 0.6 meters.

#### Validation.

Our main focus was addressing the challenge of distinguishing the real changes in large tree detection from errors introduced by a DL model. To validate the results, we used the official urban forest street tree inventory for the city of San Francisco [71]. From this inventory, we randomly selected 50 plots of one hectare in size, representing approximately 1% of the reported street tree population (1765 trees). Furthermore, we manually adjusted the GPS locations of each tree to the center of the canopy using the corresponding NAIP images. To identify large trees detected, we examined the prediction data using bounding box sizes generated during the DL model’s detection process.

The definition of a “large” tree remains unclear in the scientific literature, as no universally accepted threshold exists for distinguishing tree size categories. In this study, we classified trees into three size categories based on canopy area, derived from quartiles calculated from our validation plots (San Francisco plots test). We based our definition of large trees on the threshold established by Amati et al. [22], who classified large urban trees as those with a Diameter at Breast Height (DBH) greater than 35 cm, corresponding to an average crown radius of 3.5 meters or a crown diameter of 7 meters. Working from this definition, we identified a comparable threshold in the validation dataset from San Francisco test plots. Specifically, the 6th percentile of crown size (47 m²; see S1 Table in the supplementary material) captured trees in this criterion. While we recognize that DBH-to-crown size relationships vary by species and growing conditions, this approach provided a consistent, data-driven method to classify large trees based on their canopy.

To establish a chronological connection in predictions across the years, we leveraged the DBSCAN algorithm [72]. This algorithm facilitated grouping detections based on spatial proximity and time. Then, we incorporated site-specific variables into our model, such as the count of trees per polygon (neighborhood, city, and county), into the model to account for potential serial correlation between consecutive observations for each coordinate.

Once the large street trees were identified over time, the accuracy assessment of the number of trees was carried out. We plotted the number of large street trees on ground truth (N_g_) against detected large street trees by DL (N_dl_). Additionally, we fitted the following model to assess the relationship between N_g_ and N_dl_ and estimate the effect of the time of year the images were taken on the accuracy of N_dl_,

(d) *N*_*g*_ *~ f(N*_*dl*_*) +f(DOY) + e*

Where *f ()* are smooth spline functions [73], DOY is the day of year when the image was taken, and *e* is a Poisson error.

#### Rate of change in large street trees.

Upon establishing the real error and precision metrics, we proceeded to analyze changes in the presence of large street trees over time within the study area. To ensure that only street trees were selected, we used OpenStreetMap [74] data to clip the boundaries of houses and buildings from the detected trees. Once, the detections were validated and corrected over time (*DOY influence (d)*) the assessment of the large tree rate of change involved measurements obtained from two consecutive years:

(e) Rate of change in large street trees = [number of trees (i+1) – number of trees (i)]/[year(i+1) – year(i)]

To provide deeper context and elucidate the spatial dynamics of large street tree changes, we associated the detection outcomes for large street trees with geographical information spanning varying scales. This involved associating the validated detection outcome geographically with different levels such as track, city, and county.

### Modelling the relationship between rate of change in large tree presence, large tree per capita, canopy cover of large trees and socioeconomic variables

To ensure comparability across spatial units of different sizes, population-based variables such as white self-identified population and family’s density were normalized by census tract area (e.g., expressed as proportion or density, respectively). The values, denoted as s*(X)* in equation (f), represent smooth functions of the explanatory variables detailed in Table 2:

(f)
$y=\;bo+s\left(X_{1}\right)+\ldots +s\left(X_{k}\right)+Doy+e$

where:

*y* is one of the dependent variables (rate of change on large tree presence, large trees per capita, canopy cover of large trees); bo represents the model intercept; *X*_*1...k*_ are the independent variables; e represents a Gaussian error.

These functions were estimated using a generalized additive regression model with spline functions, as outlined by Wood et al. [73], to estimate the effects of the various explanatory variables on rate of change in number of large street trees per tract, canopy cover (percentage by tract), and trees per capita (Summary statistics for these variables are provided in S2 Table and S1 Fig). The estimation was done within the open-source R statistical package [75]. To assess the goodness of fit of the model and evaluate the amount of variability accounted for by the explanatory variables, we utilized plots comparing observed versus predicted values, specifically in the form of reliability diagrams. Additionally, R-square values were employed as a quantitative measure to gauge the proportion of variability explained by the independent variables.

## Results

The results are organized showing the results of the training and validation process of our DL models. Additionally, the following section is divided according to the three dependent variables analyzed: rate of change in large street tree presence, large street trees per capita, and canopy cover of large street trees. Within each section, we report findings at multiple spatial aggregation levels, starting from the neighborhood (NTAs), followed by the city, and concluding at the county level.

### Training process of the DL model

During the validation process we utilized the San Francisco plot information that was manually interpreted using the seven NAIP imagery datasets, with the assistance of the official urban forest street tree inventory to test the model. The overall detection accuracy was 75%, with precision and recall of 90% and 81%, respectively (based on the confusion matrix – Table 3).

**Table 3:** Confusion matrix of the model test process in San Francisco plots. TP: true positive; FP: false positives; FN: False negatives; TN: True negative.

	Trees Predicted	Background Predicted
Trees Ground-truth	TP 0.81	FN 0.19
Background Ground-truth	FP 0.08	TN 0

### Validation of the temporal series

**Fig 2:**
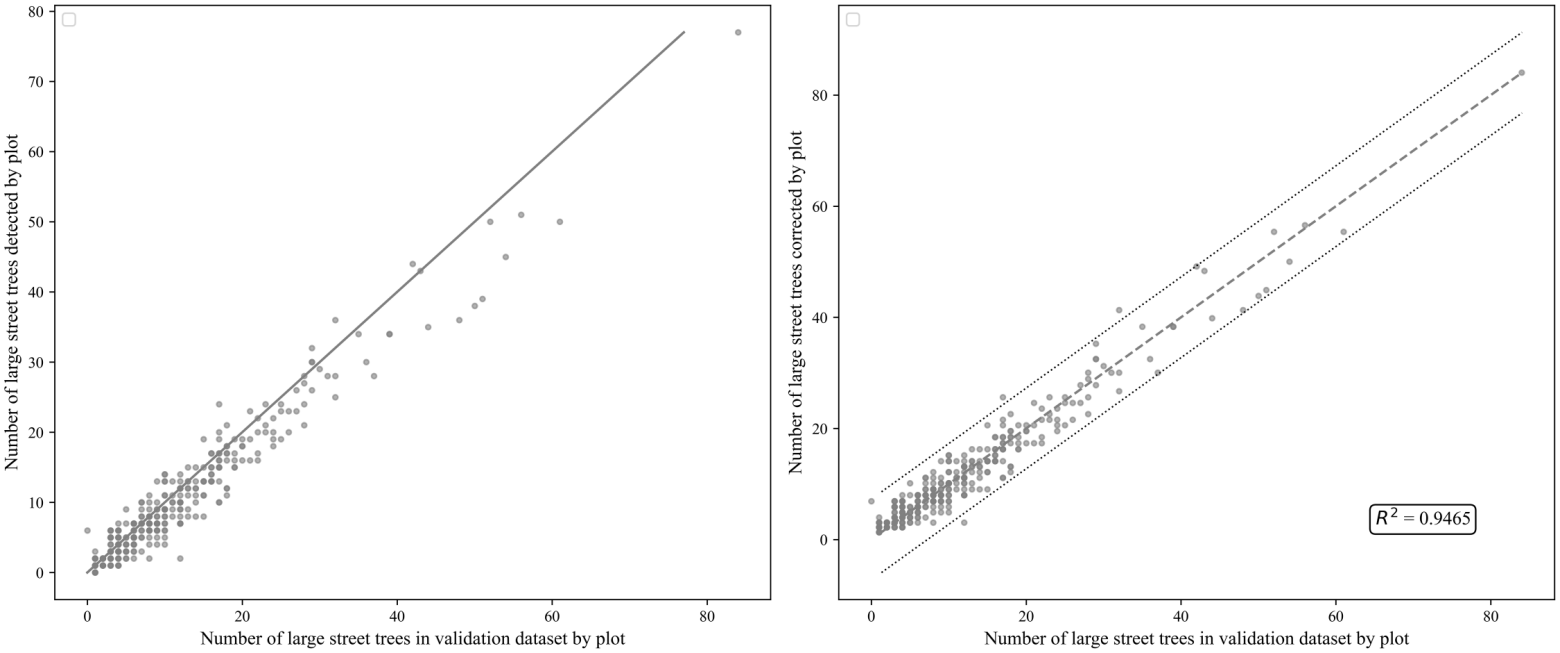
Left panel: Predicted number of large streets trees vs ground truth number of large street trees by plot. Right panel: Predicted number of large street trees vs ground truth number of large street trees by plot vs the correction with the statistical model. Dash line indicates variability.

The correlation between the number of trees detected by DL and the ground data was 0.97 with an R^2^ of 94%. However, the DL values seem to underestimate the true ground values for plots with the number of trees greater than 40 (Fig 2 left panel). The corrected values, using the estimates from model *(d)* were able to correct this bias (Fig 2 right panel).

### Spatiotemporal analysis

**Fig 3:**
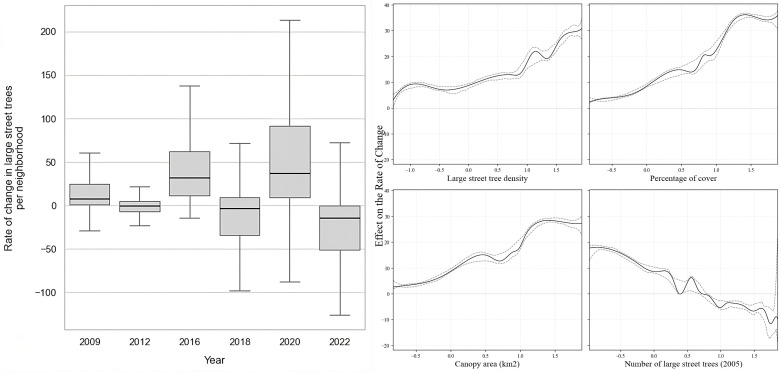
Left: Variation of the rate of change of large trees per neighborhood by year. Right: Estimated effect of variables (large street tree density, percentage of cover, canopy area and, number of trees) on the rate of change of large trees per neighborhood by year. The dashed line indicates variability.

After accounting for the negative bias, indicating an underestimation of 1.36 trees per unit (Fig 2 Right panel), at the neighborhood (NTA) level, we found a high variation in the average rate of change in large trees per year across the values for the explanatory variables (Fig 3, Left panel). The final models explained approximately 33% of the variability in the rate of change of large street trees, 40% in trees per capita, and 37% in canopy cover, indicating that the selected variables account for a substantial portion of the variation across these outcomes. The mean rate of change in large trees tends to increase as urban large street tree density and canopy cover of large street trees increase. Conversely, it tends to decrease in response to variables such as percentage of cover and the number of large street trees present in the area in the year 2005 (Fig 3).

**Fig 4:**
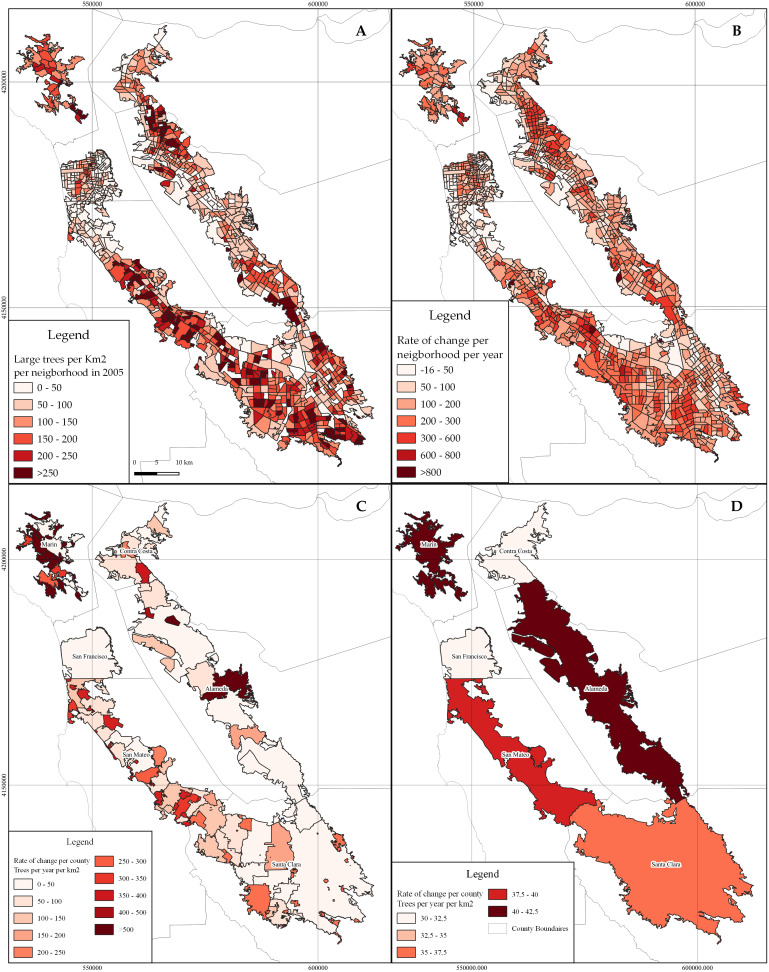
A. Map of the normalized number of large street trees by km2 in 2005 B: Normalized total rate of change (trees per year) on large street trees by neighborhood. C: Normalized total rate of change (trees per year) on large street trees by city. D: Normalized total rate of change (trees per year) on large street trees by county in the San Francisco Bay Area.

The initial number of large street trees per neighborhood (Fig 4A) in the year 2005 was mapped to determine whether the rate of change was influenced by the initial presence of them in neighborhoods or by the subsequent planting or maintenance efforts in neighborhoods with fewer large trees. As a result, we have identified a noticeable positive trend in the rate of change of large street trees, as indicated in the map (Fig 4B). This trend is particularly pronounced in regions like San Mateo County and the northwestern part of Santa Clara County. On the other hand, a significant portion of neighborhoods, accounting for at least 7% of the total, exhibit a negative trend. These areas are predominantly concentrated in counties such as the southeastern region of Santa Clara (city of San Jose), San Francisco, and Alameda (city of Oakland).

At the city level (Fig 4C), we observed a positive rate of change of large street trees in most areas, except in the city of Saratoga, where the rate of change showed a negative value, indicating a decrease of −0.2 large street trees per square kilometer per year. Some trends were observed in northwest cities in Santa Clara County, which include Stanford, Silicon Valley, and Palo Alto. In Alameda County, there were variations among cities; for instance, Oakland had a rate of change of 7 large street trees per square kilometer per year, while the city of Alameda, despite its smaller area, had a rate of change of 142 large street trees per square kilometer per year. Finally, at the county level (Fig 4D), all have had an overall positive rate of change. San Francisco and Contra Costa presented the lowest rate of change with fewer than 32 large street trees per square kilometer per year. Meanwhile, Alameda and Marin Counties exhibited the greatest rate of change with an overall difference of over 40 large street trees per square kilometer per year.

**Fig 5:**
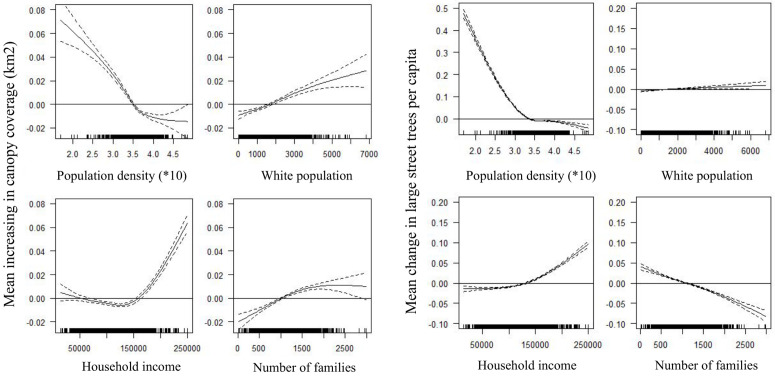
Estimated effects of social variables on average canopy coverage per neighborhood (left panel) and the average large street trees per capita (right panel). The dash area indicates the data variability.

According to the GAM results for the canopy cover model and trees per capita, population density was positively associated with changes at the neighborhood level (Fig 5).

On average, neighborhoods with a low population (less than 2000 inhabitants) exhibit an increase in canopy cover exceeding 0.05, consequently resulting in a higher mean change in large street trees per person. Conversely, neighborhoods with over 3,200 inhabitants show a negative trend in terms of the mean values for these variables. Simultaneously, mean household income demonstrates a positive correlation with the mean change in large street trees per capita, with a consistent increase corresponding to mean income levels. Concerning canopy cover, neighborhoods with lower incomes exhibit a positive mean increase, while those in the middle-income range (from 50,000–150,000 USD/year) show a negative trend in mean increase in canopy cover. Subsequently, neighborhoods with higher mean income experience a substantial increase in both the change in large street trees per capita and increasing canopy cover. Regarding the racial demographic variables, our analysis revealed a positive correlation between white population and both the mean increase in canopy cover and large street trees per capita.

## Discussion

In our study, we used the pretrained Convolutional Neural Network (CNN) specialized in canopy detection in RGB images [43] to map large street trees within the urban areas of six counties in the San Francisco Bay Area. This CNN was applied to monitor the spatiotemporal variation of these trees spanning a 17-year timeframe. We achieved an overall accuracy of 81% for large street trees detection using NAIP images of different spatial resolutions (1 and 0.6 meters) after a short training process. Our precision and recall outperformed those reported by Amanti et al. [22], who employed a similar approach in analyzing large urban trees situated on private lands. They achieved a precision of 84% and a recall of 37%. However, our level of performance is consistent with that reported by Wen et al. [76], who achieved an accuracy of 86% when mapping canopies in hyperspectral submeter images using a patch-level approach. Our result is also comparable to studies that used multiple data sources. For instance, Wu et al. [77] achieved an overall accuracy of 83% when mapping urban trees using Airborne Laser Scanning (ALS) and Terrestrial Laser Scanning (TLS) data. However, it is important to note that these studies primarily focused on assessing the predictive capacity of mapping models for single-year images with high-resolution data.

Second, the spatial resolution of our images, 0.6 and 1 meter, made it difficult to map the smallest trees (canopy < 2 meters), for which some authors recommend having spatial resolution under 50 cm [39,78,79]. To mitigate any negative impact of spatial resolution, we adjusted the window size as recommended by Velasquez-Camacho et al. [43], using windows of 250 and 350 pixels for spatial resolutions of 0.6 and 1 meter, respectively. We observed that the DOY, when the images were captured, had a notable influence on the detection of urban street trees.

Changes in the vegetative growing seasons, including leaf growth and fall, are significantly influenced by the DOY. Numerous studies have investigated the impact of DOY on foliage consolidation, and while species-specific variations exist, urban environments often exhibit notable seasonal patterns, affecting tree responses in a similar manner [80,81]. These variations in seasons can have implications for the detection of trees [82]. For instance, images captured in early May (DOY = 139), such as those from 2012 or 2022, tended to result in an overestimation of large trees. In contrast, images from 2005 or 2018, taken in early September, led to an underestimation. As noted by Alonzo et al. [83] in their study, total leaf growth in urban areas typically occurred around DOY = 120, while the leaf-off season might start around DOY = 300. These seasonal changes come with variations in leaf color, brightness, and shape. Additionally, the end of the growing season can be influenced by summer and fall air temperatures Alonzo et al. [83] found that the timing of foliage loss for certain genera could differ by up to 20 days between years. For instance, *Prunus sp*. began losing its foliage on DOY = 305 in 2019 and DOY = 280 in 2020. This effect is particularly relevant for numerous notable trees in these cities, which happen to be deciduous species like *Platanus x hispanica* or *Prunus sp.* [84,85]. These deciduous trees undergo significant changes in their foliage over time, and this variability subsequently impacts the accuracy of canopy detection, particularly when considering our threshold for categorizing trees as large trees (7 meters of canopy diameter). Nonetheless, our analysis accounts for this effect and adjusts the results to mitigate the bias introduced by the date of image acquisition.

Indeed, apart from the foliage variations, the timing of pruning and cutting activities can introduce discrepancies in tree detection and mapping based on the timing of the aerial imagery. For instance, San Francisco requires a pruning prerequisite that each tree within the city must undergo pruning at intervals ranging from three to five years, with an average removal rate of 25% of the canopy [86]. Contrary, Palo Alto implement a more frequent pruning regimen, with a pruning cycle as short as every two years [87]. In contrast, San Jose pruning cycles spanning 5–7 years [84]. It is imperative to acknowledge that these varying pruning schedules and the associated tree removal practices can substantially modify the visual characteristics of trees and their canopies [88], as well as affect the overall health of the canopy, rendering this as an important consideration in the context of tree detection.

These pruning schedules are closely related to the available budget allocated for urban forestry in different cities [89]. The presence of large and healthy street trees is influenced by a combination of biological factors like survival rates and or social initiatives such as planting, care, and maintenance [6,89]. For instance, Oakland ArcGIS StoryMap provided by City of Oakland [90] not only reports a 21% canopy cover, which is notably higher compared to surrounding cities, but also emphasizes the challenge of canopy cover loss due to inadequate funding for the city’s tree care program. This loss amounts to approximately 3.4% of canopy cover between 2014 and 2020. This aligns with our findings, as we observed that Alameda County, particularly in the Oakland region, exhibits a low rate of change in street trees canopy cover over the analyzed period. A similar situation can be observed in San Jose, where we identified a minimal rate of change of 181 large trees per year. This aligns with the report from the San Jose Community Forest Management Plan, which discusses an 11.8% decrease in canopy cover in the last decade in San Jose [84].

Furthermore, the pronounced income disparities among different cities and neighborhoods within the region [91] coupled with variations in the quality and quantity of urban trees, have been well-documented. Our findings from the model analyzing the rate of change in large tree presence suggest that areas with lower income levels and higher residential densities tend to experience lower or even negative rates of change, especially at the neighborhood scale. These results are consistent with those reported by [10] that found that the neighborhoods categorized as “A-graded neighborhoods” in Baltimore are nine times more likely to contain a large tree than “D-graded neighborhoods”. There is substantial evidence supporting the relationship between large tree canopies and higher-income neighborhoods [6,92,93]. Our research corroborates this trend, highlighting a strong connection between canopy cover increasing and household incomes, particularly in areas with incomes over USD 150,000. We observed that areas such as Silicon Valley or Palo Alto boast a greater number of large street trees compared to lower-income areas, such as the northern part of San Mateo County, and Vallejo area.

Additionally, our research found that racial segregation also plays a role in EJ, as areas with a higher white population tend to have a greater presence of canopy cover from large street trees. Similarly, Casey et al. [94] reported that census tracts with a higher percentage of racial/ethnic minorities, in contrast to those with a higher percentage of White residents, exhibited lower greenery levels in 2001 and experienced more substantial greenery loss between 2001 and 2011. Research by Wilson [95] and Rigolon [96] are in the same vein. Both noted the impact of race and ethnicity on canopy cover and the presence of trees in neighborhoods. Wilson [95] highlighted that neighborhoods with ethnic and minority populations have historically received less investment in environmental amenities, including trees. Similarly, Rigolon [96] discovered that predominantly White municipalities across the United States tend to have higher-quality parks compared to Black and Latino municipalities.

This study carries significant implications for decision-making and urban planning in the San Francisco Bay Area. Our findings provide valuable insights that can guide decision-makers and stakeholders in identifying the need for targeted interventions. These interventions may include reforestation initiatives, tree planting programs, maintenance of trees post-planting, or the implementation of stricter regulations regarding tree removal, particularly in neighborhoods facing greater vulnerability. Furthermore, it is noteworthy that California, as outlined by Urban forestry: Statewide Strategic Plan of California [97], has set a goal of increasing canopy cover by 10% by 2035, with a specific focus on disadvantaged and low-income communities, as well as areas with limited canopy coverage. In the same vein, the city of San Jose [98], highlights the goal of increasing the canopy cover in cities and targeting low-income areas (key action 3.16). However, achieving this goal requires more than simply planting new trees; it also entails preserving and expanding the presence of large, mature trees. While tree planting is a crucial component of this effort, the protection and growth of existing mature trees are equally vital for effectively enhancing urban canopy cover.

Our results highlight how tree-related indicators, such as trees per capita and canopy cover, intersect with demographic and socioeconomic variables, supporting their use as proxies for environmental justice. By combining these indicators with spatial and temporal analyses, we provide empirical evidence of persistent inequalities in access to the ecosystem services provided by large urban street trees.

### Limitations and future research

While our study demonstrates the potential of remote sensing data and artificial intelligence algorithms to monitor large street tree dynamics, it is not without limitations. These technologies offer a powerful means of monitoring and understanding changes in urban forestry, which is crucial for effective urban planning, environmental management, and the pursuit of green justice. For future research, we suggest incorporating techniques that enhance the selection of large trees by considering both tree height and canopy size. This approach will help to reduce the misclassification of ground cover and shrubs as trees, thereby improving the accuracy of tree identification. However, one key constraint is the exclusive focus on street trees, typically managed by local governments, which exclude large trees located in backyards and other private areas. This limits the comprehensiveness of our canopy assessments, as a substantial portion of urban tree cover falls outside the public domain. Future research should explore the integration of backyard trees into canopy monitoring frameworks, leveraging very high-resolution imagery or 3D datasets to capture trees on private parcels.

Additionally, further studies could focus on inter-city comparisons of street tree temporal dynamics and management regimes. Such comparisons would be particularly those that incorporate equity-focused practices aimed at increasing tree numbers and canopy cover in disadvantaged areas (See supplementary material S4 Fig). To evaluate whether the residuals of our model exhibited spatial clustering, we performed a Moran’s I test at three spatial scales: census tract, city, and county (See supplementary material S3 Table). The results confirmed the presence of strong spatial clustering at both the city level (Moran’s I = 0.58, p = 0.0336) and county level (Moran’s I = 0.70, p = 0.001). These findings indicate that, although the tract-level patterns are more heterogeneous, there is a clear spatial structure in our model residuals at broader spatial units. This spatial dependence suggests that future analyses should consider spatially explicit models, such as spatial lag models or geographically weighted regression, to improve inference and better capture neighborhood effects on large street tree dynamics. By integrating these quantitative approaches with qualitative data, such as administrative records on street tree removals and interviews with municipal arborists, future research can provide a more holistic understanding of the mechanisms driving street tree dynamics. These combined methods will offer a comprehensive framework for advancing urban forestry management, with an emphasis on promoting environmental justice and equitable access to urban green spaces.

By integrating these quantitative approaches with qualitative data, such as administrative records on street tree removals and interviews with municipal arborists, future research can provide a more holistic understanding of the mechanisms driving street tree dynamics. These combined methods will offer a comprehensive framework for advancing urban forestry management, with an emphasis on promoting environmental justice and equitable access to urban green spaces.

## Conclusions

This research aims to assess the capacity of multitemporal remote sensing data and artificial intelligence techniques to quantify the presence of large street trees in urban areas. Through an evaluation of cities in the San Francisco Bay Area, we have identified these technologies as a significant advancement in the detection, monitoring, and analysis of urban street trees. This approach has enabled us to gain new insights into the temporal changes in tree populations and their correlation with socio-demographic variables. This study successfully addresses various key aspects, including the analysis of time series data, tree mapping, change tracking, and equity in green space access within cities. Our findings reveal uneven patterns of large street tree loss and gain, with lower-income and racially diverse neighborhoods showing slower growth or even declines in tree presence. These disparities underscore the need for targeted urban forestry interventions that prioritize environmental equity.

## Supporting information

S1 Table Canopy size (m^2^) distribution by percentiles in validation plots in San Francisco City based on deep learning model predictions. (DOCX)

S2 Table Summary statistics (mean, median, standard deviation [SD], standard error [SE], minimum, and maximum) for the main dependent and independent variables used in the models. Variables include tree-related metrics, sociodemographic indicators, and population measures, aggregated across all years and census tracts.
(DOCX)

S3 Table Global Moran’s I statistics calculated on the residuals of the GAM model at three spatial scales: census tract, city, and county. Spatial weights were defined using a k-nearest neighbors’ approach (k = 6).
(DOCX)

S1 Fig Spatial distribution of urban street trees and socioeconomic characteristics across census tracts in the San Francisco Bay Area. (TIF)

S2 Fig Temporal trends in three key urban forestry metrics: trees per capita, percentage of cover, and rate of change in large street trees, across three census tracts representing distinct sociodemographic profiles: high income (GEOID: 6081612800), high percentage of White population (GEOID: 6085503504), and low income and low percentage of White population (GEOID: 6075012502). (TIF)

S3 Fig Examples of change over time of detected trees. Base map imagery (NAIP) from the USDA Farm Production and Conservation – Business Center, Geospatial Enterprise Operations, used under public domain guidelines.
(TIF)

S4 Fig Examples of tree removal dynamic. Base map imagery (NAIP) from the USDA Farm Production and Conservation – Business Center, Geospatial Enterprise Operations, used under public domain guidelines.
(TIF)
