# Supplementary material for: Monitoring temporal changes in large urban street trees using remote sensing and deep learning
Source: PLoS One. 2025 Jun 26;20(6):e0326562. doi: 10.1371/journal.pone.0326562 (PMC12200653; doi:10.1371/journal.pone.0326562)
Supplement: S1 Table — (DOCX) [file pone.0326562.s001.docx]

Table 1S: Canopy size (m^2^) distribution by percentiles in validation plots in San Francisco City based on deep learning model predictions.

| Percentile | Bounding box area (m^2^) | Tree category |
| --- | --- | --- |
| mean | 72,440 |  |
| std | 107,152 |  |
| min | 3,1673 |  |
| 1% | 5,6275 | Small tree |
| 10% | 11,0026 |  |
| 20% | 15,0672 |  |
| 30% | 19,6782 | Medium tree |
| 40% | 26,3574 |  |
| 50% | 35,0166 |  |
| **60%** | **47,0307** | **Threshold**  **for large tree** |
| 70% | 66,1137 |  |
| 80% | 98,3743 |  |
| 90% | 173,864 |  |
| max | 1598,14269 |  |
