# Supplementary material for: Monitoring temporal changes in large urban street trees using remote sensing and deep learning
Source: PLoS One. 2025 Jun 26;20(6):e0326562. doi: 10.1371/journal.pone.0326562 (PMC12200653; doi:10.1371/journal.pone.0326562)
Supplement: S2 Table — Variables include tree-related metrics, sociodemographic indicators, and population measures, aggregated across all years and census tracts. (DOCX) [file pone.0326562.s002.docx]

Table 2S Summary statistics (mean, median, standard deviation [SD], standard error [SE], minimum, and maximum) for the main dependent and independent variables used in the models. Variables include tree-related metrics, sociodemographic indicators, and population measures, aggregated across all years and census tracts.

|  | **Mean** | **Median** | **SD** | **SE** | **Min** | **Max** |
| --- | --- | --- | --- | --- | --- | --- |
| **Tree per capita** | 0.088 | 0.063 | 0.093 | 0.001 | 0 | 1.045 |
| **Percentage of cover (%)** | 4.11 | 3.33 | 3.16 | 0.042 | 0.001 | 19.84 |
| **Rate of change (number of large street tree)** | 11.33 | 4.5 | 62.022 | 0.819 | -529.5 | 572.5 |
| **Household income (dollar)** | 110851 | 105561 | 46129.92 | 609.61 | 14976 | 250001 |
| **Percentage of White population** | 0.637 | 0.655 | 0.225 | 0.003 | 0.083 | 0.998 |
| **Total Population** | 4511.58 | 4428.10 | 1523.61 | 217.02 | 50.86 | 10288 |
| **Total families** | 1042.01 | 1004.0 | 412.34 | 6.05 | 17 | 2980 |
| **Number of Large Street Trees** | 393.42 | 277 | 399.11 | 5.272 | 1 | 3633 |
