# Supplementary material for: Monitoring temporal changes in large urban street trees using remote sensing and deep learning
Source: PLoS One. 2025 Jun 26;20(6):e0326562. doi: 10.1371/journal.pone.0326562 (PMC12200653; doi:10.1371/journal.pone.0326562)
Supplement: S3 Table — Spatial weights were defined using a k-nearest neighbors’ approach (k = 6). (DOCX) [file pone.0326562.s003.docx]

Table 3S. Global Moran’s I statistics calculated on the residuals of the GAM model at three spatial scales: census tract, city, and county. Spatial weights were defined using a k-nearest neighbors’ approach (k = 6).

| **Scale** | **Moran’s I** | **p-value** | **Expected I under null** |
| --- | --- | --- | --- |
| **Global** | 0.2346 | 0.0010 | -0.0002 |
| **Tract** | -0.1942 | 0.2903 | -0.2000 |
| **City** | 0.5303 | 0.0466* | -0.0305 |
| **County** | 0.7227 | 0.001** | -0.0023 |
