# Supplementary figures and images for: Monitoring temporal changes in large urban street trees using remote sensing and deep learning
Source: PLoS One. 2025 Jun 26;20(6):e0326562. doi: 10.1371/journal.pone.0326562 (PMC12200653; doi:10.1371/journal.pone.0326562)

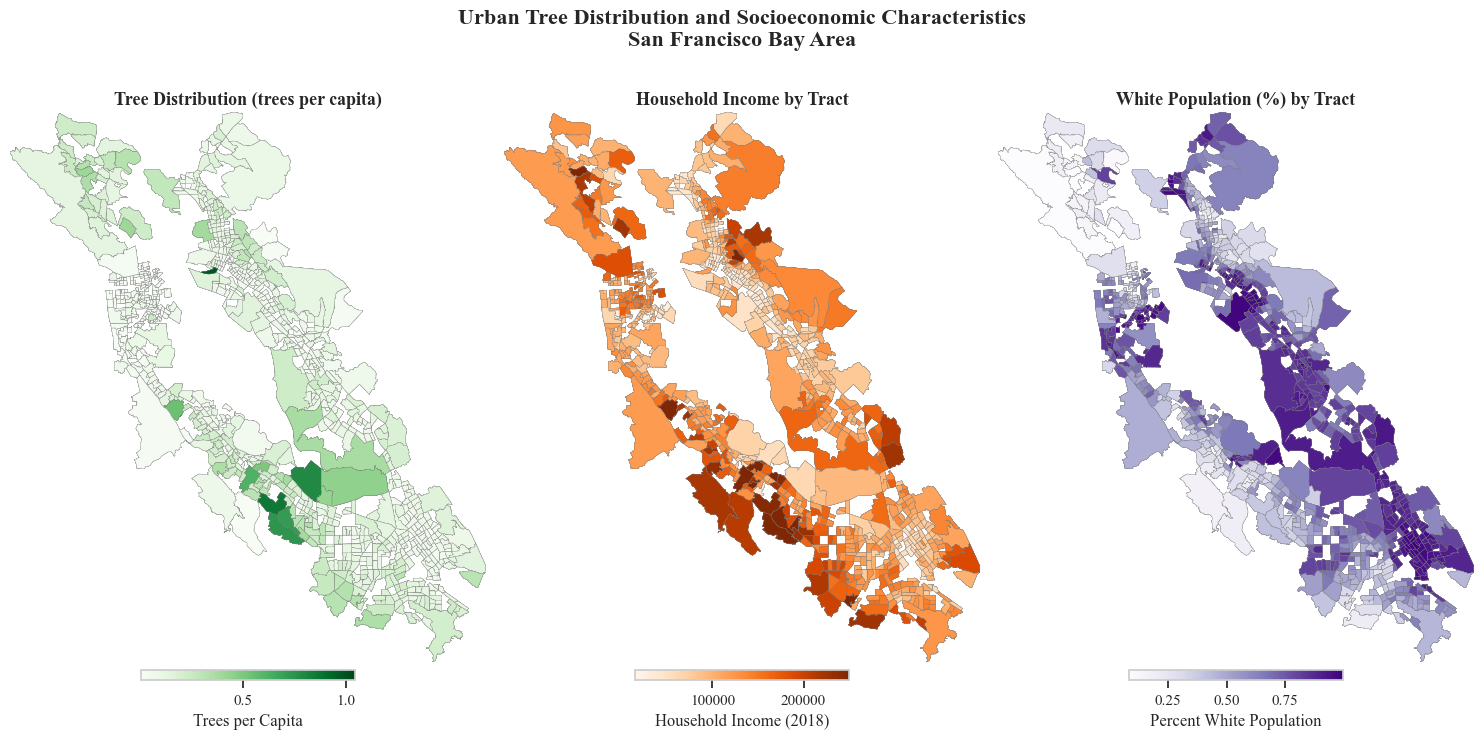

Supplement: S1 Fig — (TIF) [file pone.0326562.s004.tif]

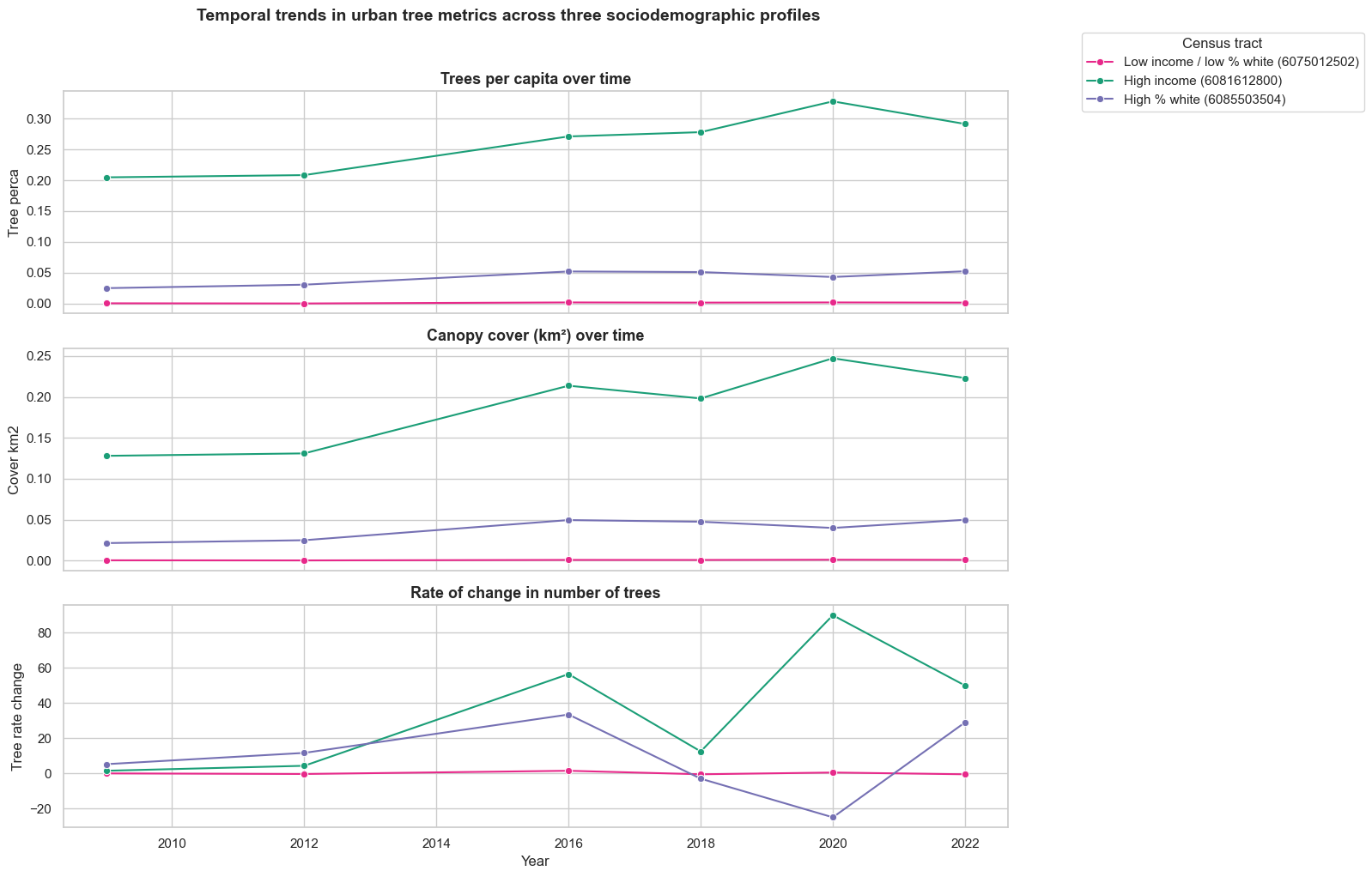

Supplement: S2 Fig — (TIF) [file pone.0326562.s005.tif]

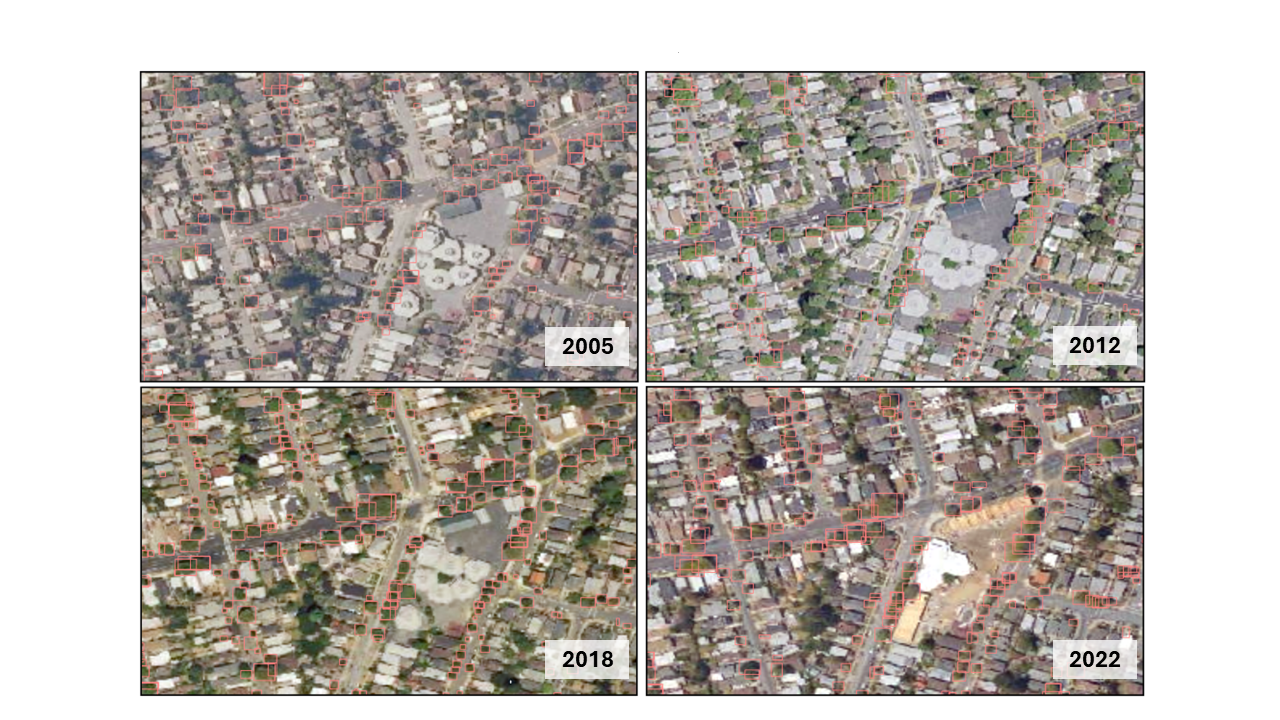

Supplement: S3 Fig — Base map imagery (NAIP) from the USDA Farm Production and Conservation – Business Center, Geospatial Enterprise Operations, used under public domain guidelines. (TIF) [file pone.0326562.s006.tif]

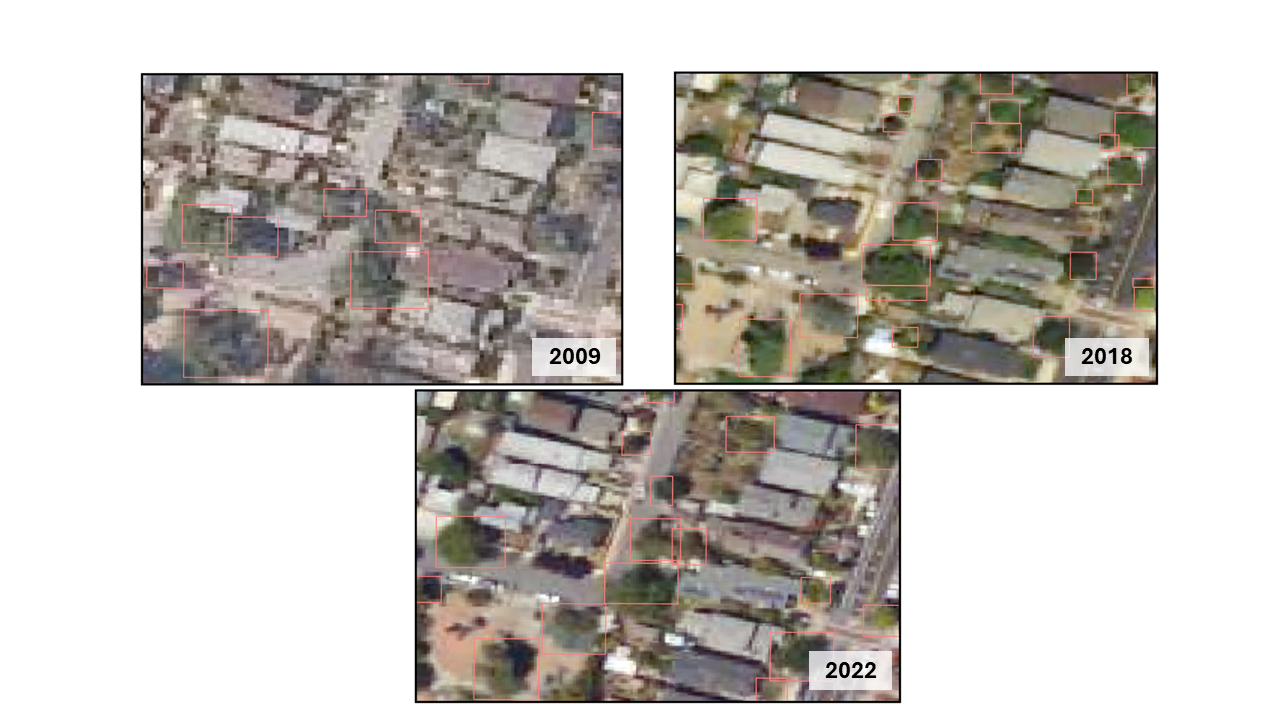

Supplement: S4 Fig — Base map imagery (NAIP) from the USDA Farm Production and Conservation – Business Center, Geospatial Enterprise Operations, used under public domain guidelines. (TIF) [file pone.0326562.s007.tif]
